# Omentin-A Novel Adipokine in Respiratory Diseases

**DOI:** 10.3390/ijms19010073

**Published:** 2017-12-28

**Authors:** Yan Zhou, Bo Zhang, Caixia Hao, Xiaoting Huang, Xiaohong Li, Yanhong Huang, Ziqiang Luo

**Affiliations:** Department of Physiology, Xiangya School of Medicine, Central South University, Changsha 410008, China; 166511019@csu.edu.cn (Y.Z.); zhangboxy@csu.edu.cn (B.Z.); haocaixia1122@csu.edu.cn (C.H.); huangxiaoting@csu.edu.cn (X.H.); li1989@csu.edu.cn (X.L.); 802224@csu.edu.cn (Y.H.)

**Keywords:** omentin, adipokines, adipose tissue, respiratory diseases, MPM, asthma, OSAS, PAH, ARDS, COPD

## Abstract

Adipokines, secreted by the adipose tissue, are extensively involved in the regulation and maintenance of various physiological and pathological processes, including insulin sensitivity, energy expenditure, glucose and lipid metabolism, inflammatory activity, neuroendocrine activity, immunity, cancer, homeostasis, angiogenesis, cardiovascular function, breeding and bone metabolism, and all functions of the endocrine-reproductive system axis. Omentin is a recently identified adipokine, which has become a research hotspot due to its pleiotropic effects on various diseases. However, the specific receptor for omentin has not been identified so far. In this study, we report that omentin levels fluctuate in various diseases. In addition, we have focused on the pleiotropic roles of omentin in pulmonary diseases, as it may act as a biomarker for malignant pleural mesothelioma (MPM) and is related to disease severity. Omentin may play significant roles in other pulmonary diseases, such as asthma, obstructive sleep apnea syndrome (OSAS), pulmonary arterial hypertension (PAH), acute respiratory distress syndrome (ARDS), and chronic obstructive pulmonary disease (COPD). This review summarizes the advances in current knowledge and future trends, which may provide a concise and general view on omentin and its effects on pulmonary biology.

## 1. Introduction

Various adipokines, including adiponectin, adipsin, apelin, chemerin, fibroblast growth factor 21, interleukin-6 (IL-6), leptin, retinol-binding protein 4, osteonectin, omentin, plasminogen activator inhibitor-1, progranulin, resistin, tumor necrosis factor (TNF), vaspin, and vistafin are derived from the adipose tissue [1,2].

Adipokines participate in various physiological and pathological processes including insulin sensitivity, energy expenditure, glucose and lipid metabolism, inflammatory activity, neuroendocrine activity, immunity, cancer, homeostasis, angiogenesis, cardiovascular function, breeding and bone metabolism, and all functions of the endocrine-reproductive system axis [2,3,4]. The majority of adipokines, such as TNF-α and IL-6, pose adverse effects and aggravate the severity of diseases [5,6]. On the contrary, a few of these, such as adiponectin and omentin, are “good” adipokines [7,8]. Omentin is an important molecule that connects organs with adipose tissue and exerts extensive protective effects [9,10]. Pulmonary diseases are devastating disorders with high morbidity and mortality, and understanding the cellular and molecular mechanisms of omentin function in these diseases is a key step towards improving research on pulmonary disease biology. Therefore, this review focuses on the latest advances in omentin biology and its effects on the development and progression of pulmonary diseases.

## 2. The Structure and Development of Omentin

Omentin is a novel hydrophilic adipokine of 313 amino acids (35 kDa), which contains a secretory signal sequence and a fibrinogen-related domain, and appears as a glycolized trimer of 120 kDa molecular weight in its negative form [10,11]. In 2005, omentin was officially depicted in an omental fat cDNA library with Uniprot code Q8WWAO and Genbank accession number AY549722 [12]; it was initially identified in intestinal Paneth cells and endothelial cells with the name intelectin-1, intestinal lactoferrin receptor, galactofuranose binding lectin, and endothelial lectin [13,14]. However, specific receptors for omentin have not yet been identified. Omentin-1 and omentin-2 are two highly homologous isoforms with 83% amino acid identity, the genes encoding which are proximal to the 1q22–q23 chromosomal region associated with type-2 diabetes mellitus (T2DM) in many individuals [12,15]. Omentin-1 is the major circulating form with a concentration of 100 ng/mL to 1 μg/mL in human plasma, and has been more extensively studied than omentin-2 [10]. Omentin-1 is a Ca^2+^-dependent galactofuranose-binding lectin used for identifying bacterial components, and is important for defense against pathogenic bacteria [12,13]. In the following sections, we will refer to omentin-1 or intelectin-1 as omentin.

## 3. The Concentration of Omentin Fluctuates in Various Diseases

In recent years, considerable progress has been made in determining and characterizing the effects of omentin on different diseases. Based on a large number of epidemiological, clinical, and laboratory analyses, the changes in omentin levels in various diseases have been summarized in Table 1. 

Omentin levels are inversely related to obesity and positively to adiponectin levels [16]. Several studies have shown that higher omentin levels were associated with leanness or acted as a positive factors against obesity [16,17,18]. Reports also showed that pre-existing obese pregnant women had lower omentin levels in the placenta and adipose tissue than their normal weight counterparts [19]. In addition, studies indicated that the serum levels of omentin were low in patients with impaired glucose regulation, T2DM [20], gestational diabetes mellitus (GDM) [21], T2DM with ischemic heart disease [22,23], and diabetic retinopathy [24]. Recent reports showed that circulating omentin levels in women with polycystic ovary syndrome (PCOS) were significantly lower than that in normal women, independent of body mass [25,26]. Moreover, the results of a meta-analysis [27] including 1264 subjects (733 patients with PCOS and 531 controls) showed a significant decrease in circulating omentin levels in patients with PCOS. Serum omentin levels apparently increased in PCOS individuals after administration of metformin. These prompted the speculation that omentin may play a significant role in the pathogenesis of PCOS [28].

Several studies demonstrated a significant reduction in omentin levels in various cardiovascular diseases, including patients with carotid atherosclerosis [29,30,31], coronary artery disease (CAD) [32,33], heart failure [34], and dilated cardiomyopathy [35]. Another study demonstrated that omentin increased in the epicardial adipose tissue (EAT) but decreased in plasma in patients with CAD [32]. Harada explained this contradictory phenomenon by postulating that the high levels of omentin isolated from the EAT in myocardial ischemia may exert a cardioprotective effect [32]. Surprisingly, recent studies showed that omentin levels were elevated in nonalcoholic fatty liver disease (NAFLD) [36], an obesity-related disease, although obesity is associated with low levels of omentin. More studies are warranted to elucidate the mechanisms underlying these contradictory observations.

Omentin levels were reduced in certain autoimmune diseases, including psoriasis [37,38,39], rheumatoid arthritis [40,41], Behcet’s disease [42], Crohn’s disease [43], ulcerative colitis [44], chronic periodontitis [45], and acute or chronic pancreatitis [46]. Other studies demonstrated that omentin levels were highly elevated in individuals with psoriatic arthritis compared to individuals with psoriasis alone or healthy individuals [47], although the precise mechanism underlying these observations is not obvious. In contrast, other research groups demonstrated that the presence of nephritis was associated with elevated plasma omentin levels in patients with systemic lupus erythematosus (SLE) [48]. In addition, Peraire [49] demonstrated that human immunodeficiency virus/highly active anti-retroviral therapy (HIV/HAART)-associated lipodystrophy syndrome (HALS) was related to decreased omentin levels in plasma, indicating that omentin may be an important contact between HIV/HAART and fat redistribution syndromes. Furthermore, the circulating levels of omentin were significantly lower in painful temporo-mandibular disorders (TMD), which may be mediated by inflammatory pathways [50].

In addition, circulating omentin levels were also dramatically reduced in renal cell cancer [51]; however, other cancers, such as malignant pleural mesothelioma (MPM) [52], hepatic carcinoma [53], prostate cancer [54], colon and colorectal cancer [55,56], gastric cancer [57], and pancreatic adenocarcinoma [58] were associated with increased omentin levels. Omentin may have an anti-cancer effect property where omentin can influence two types of human hepatocellular carcinoma cells: HepG2 and HuH-7. Omentin significantly inhibited the proliferation and promoted apoptosis of HepG2 and HuH-7 cells via activating the Jun N-terminal kinase (JNK)-p53 signaling pathway [53]. Moreover, omentin can accelerate the apoptosis of hepatocellular carcinoma cells (HCC) by increasing the bax/bacl-2 ratio and inducing capases-3 activation [53]. Subsequently, studies were conducted to investigate the effect of omentin in the respiratory system. A study demonstrated that circulating omentin levels were also dramatically decreased in patients with acute respiratory distress syndrome (ARDS) [59]. Omentin may play an important role in defense against pathogenic bacteria, and lower omentin levels in smokers may contribute to increased susceptibility to infection [60]. However, the results obtained with patients exhibiting obstructive sleep apnea syndrome (OSAS) were controversial. Wang et al. observed that serum omentin levels were significantly lower in patients with OSAS [61], while two other groups showed that these patients had considerably higher omentin levels [62,63]. The researchers also observed that omentin levels increased in airway epithelial cells of asthmatic individuals and speculated that omentin may be involved in the pathogenesis of asthma [64,65].

The serum levels of omentin were also significantly higher in other diseases, such as liver cirrhosis [66] and anorexia nervosa [67,68], and in individuals undergoing hemodialysis with end stage renal disease [69].

## 4. Protective Effects of Omentin in Various Pathophysiological Processes

**Figure 1:**
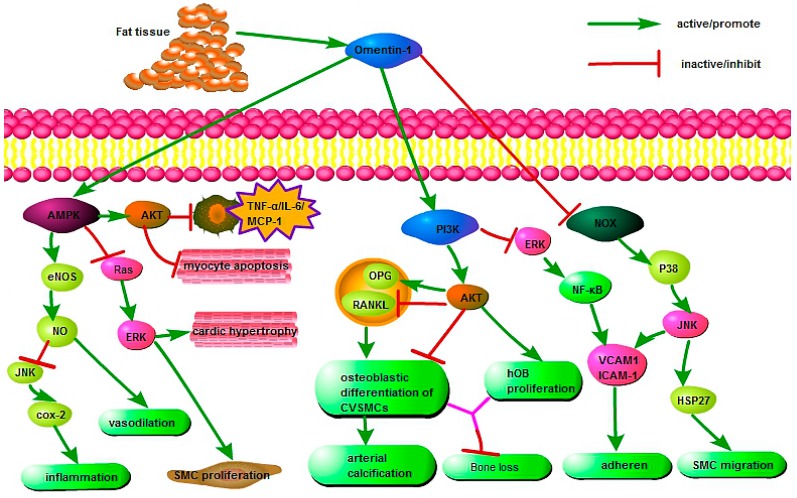
The protective mechanisms of omentin in various pathophysiological processes. AMPK, Adenosine 5′-monophosphate (AMP)-activated protein kinase; eNOS, endothelial nitric oxide synthase; COX-2, cyclooxygenase-2; ERK, extracellular regulated protein kinases; TNF-α, tumor necrosis factor-α; IL-6, interleukin-6; MCP-1, monocyte chemotactic protein-1; PI3K, phosphatidylinositol 3 kinase; AKT, protein kinase B; OPG, osteoprotegerin; RANKL, receptor activator for nuclear factor κB ligand; CVSMC, calcifying vascular smooth muscle cells; VCAM-1, vascular cell adhesion molecule-1; ICAM-1, intracellular adhesion molecule-1; NOX, NADPH oxidase; JNK, Jun N-terminal kinase; HSP27, heat shock protein 27; SMC, smooth muscle cell; NF-κB, nuclear factor-κB.

Omentin is an important component that connects organs with adipose tissue and exerts extensive protective effects via various cell signaling pathways during physiological and pathological processes (Figure 1). Yamawaki et al. indicated that omentin inhibited TNF-α-induced cyclooxygenase-2 (COX-2) expression via activation of adenosine 5′-monophosphate-activated protein kinase (AMPK), which further activated the endothelial nitric oxide synthase (eNOS)/NO pathway and blocked Jun N-terminal kinase (JNK) signaling, thereby playing an anti-inflammatory role in endothelial cells [70]. Moreover, the activation of eNOS/NO induced vasodilation in isolated blood vessels [71] and decreased agonist-induced increase in blood pressure [72,73]. Second, the omentin-induced AMPK phosphorylation can also reduce the RAS/ERK signaling cascade, accompanied by reduction of cardiac hypertrophy [74] and smooth muscle cell (SMC) proliferation [9,75]. Third, studies demonstrated that omentin can promote the AMPK/AKT pathway directly by suppressing myocyte apoptosis in acute ischemic heart injury [76] and decreasing the expression of proinflammatory mediators, including TNF-α, IL-6, and monocyte chemotactic protein-1 (MCP-1) in macrophages [77].

Moreover, omentin protected against arterial calcification by inhibiting osteoblastic differentiation of calcifying vascular smooth muscle cells (CVSMCs) via the phosphatidylinositol 3 kinase/protein kinase B (PI3K/AKT) signaling pathway [78]. This may be associated with increased production of osteoprotegerin (OPG) and decreased receptor activation for nuclear factor κB ligand (RANKL) in both CVSMCs and osteoblasts [79]. In addition, omentin promoted human osteoblast (hOB) proliferation through the PI3K/AKT signaling pathway [80]. Importantly, omentin suppressed adhesion of monocytes to TNF-α-activated endothelial cells by inhibiting ICAM-1 and VCAM-1 expression via PI3K-AKT signaling and by blocking the ERK/NF-κB pathway [81].

Furthermore, omentin inhibited VCAM-1 expression in vascular smooth muscle cells (VSMCs) via phosphorylation of p38 and JNK at least in part through preventing NADPH oxidase (NOX)-derived superoxide production; it also restrained TNF-α-induced adhesion of U937 monocytes to isolated rat VSMCs [82]. Finally, omentin also inhibited NOX/O2-/p38/heat shock protein 27 (HSP27) pathways to prevent platelet-derived growth factor (PDGF-BB)-induced smooth muscle cell (SMC) migration, which may be related to its protective role in neointimal hyperplasia [83].

## 5. Roles of Omentin in Pulmonary Disease

### 5.1. Omentin in Malignant Pleural Mesothelioma (MPM)

Malignant pleural mesothelioma (MPM) is an uncommon but lethal tumor. The effect of current treatment on MPM is poor, and surgery is most effective for patients in early stage MPM. Unfortunately, because of the lack of characteristic clinical symptoms, radiographical features, and specific diagnostic markers for MPM, diagnosis of MPM at the early stage is difficult. The median survival after radical surgery or chemotherapy is only 9–12 months [84,85].

A study suggested that both the mRNA and protein levels of omentin increased (>129-fold increase) in mesothelioma tumors and cell lines [52,86]. Epithelioid-type MPMs, but not lung adenocarcinomas with pleura invasion or adjacent reactive mesothelial cells, can be stained with anti-omentin antibodies. Moreover, patients with MPM possessed higher omentin levels than patients with lung cancer and pleural effusion; omentin concentration in pleural effusions of patients with MPM (MPM) is about 3000 ng/mL, while in lung cancer (LC), tuberculosis (TB), and pleuropneumonia (PP), the average levels of omentin are about 300, 250 and 650 ng/mL [86], respectively. Studies demonstrated that except for some mucinous adenocarcinomas, omentin was not expressed in other cancers, such as biphasic synovial sarcoma, breast carcinoma, colon adenocarcinoma, epithelioid angiosarcoma, epithelioid hemangioendothelioma, gastric adenocarcinoma, lung cancer, ovarian adenocarcinoma, renal cell cancer, and urothelial cancer [87]. Fortunately, mucinous adenocarcinomas can be easily distinguished from MPM by other methods [87]. 

Therefore, these results suggested that omentin in the pleural effusion could be used as a specific diagnostic marker for distinguishing epithelioid-type MPMs from other carcinomas because of its specificity and the simplicity of pathological assessment. 

### 5.2. Omentin in Asthma

Asthma is a disease characterized by T-helper type 2 (Th2) allergic airway inflammation, airway hyperresponsiveness, mucus overproduction, and peribronchial fibrosis. Kuperman et al. demonstrated that omentin-1 expression was enhanced in ovalbumin (OVA) allergic mice and IL-13-overexpressing mice [64]. They also showed that omentin mRNA was significantly upregulated in the airway epithelial cells from asthmatic individuals [64]. IL-13 treatment increased omentin mRNA levels in cultured primary human bronchial epithelial cells and the mouse airway in vivo [88]. Furthermore, omentin was up-regulated in the sputum of subjects with asthma [89]. A study demonstrated that a single-nucleotide polymorphism in omentin was related to increased asthma risk [90]. These observations indicated that omentin may play an important role in the pathogenesis of asthma. 

Studies of mouse asthma models showed that IL-13 was necessary for allergen-induced airway inflammation, airway hyper responsiveness, and mucus production [91,92]. IL-13 prompts the production of MCP-1 and MCP-3 in mouse lung tissue and human bronchial epithelial cells through the activation of p38, APK, ERK, and JAK-2 [93]. Studies showed that MCP-1, MCP-3 and omentin levels were rapidly increased after access to airway allergens. Importantly, omentin was mainly expressed in airway mucous cells [94]. Gu et al. [94] also showed that the increase in MCP-1 and -3 mRNA levels were completely inhibited by omentin shRNA and galactose, which binds to omentin and inhibits its function [95]. Moreover, inhibition of omentin expression also decreased the levels of secreted MCP-1 and -3 in vivo. In addition, eosinophil counts in bronchoalveolar lavage fluid (BALF) and inflammatory cell infiltration around airways in OVA-challenged mice were reversed by omentin knock-down [94]. These data indicated that omentin was essential for IL-13-induced MCP-1 and MCP-3 expression in mouse lung epithelial cells and it promoted allergic airway inflammation. Furthermore, IL-25 and IL-33 levels were enhanced in asthma during innate immune response to allergens, and they play important roles at the onset of allergic inflammation in asthma [96,97]. Studies demonstrated that omentin was involved in allergen-induced IL-25 and IL-33 production in asthma [98]. These results indicated that omentin may participate in the pathogenesis of airway inflammation, airway hyper responsiveness, and mucus overproduction in asthma.

Furthermore, omentin may be a component of airway mucus. This may be conducive to forming pathologic mucus and defending against microbes. A study showed that omentin was a goblet cell protein, which is secreted with mucus into the intestinal lumen [87]. In addition, other studies in the intestine have suggested that mucin–omentin interactions may alter the biophysical properties of mucus [99]. As we know, bronchial inflammation can cause hyperplasia of goblet cells and enhances the production of mucus [100]. In that way, prominent immunostaining for omentin in asthmatic mucus and the high concentrations of omentin in sputum of patients with acute severe asthma corroborate these observations [89]. Moreover, omentin can also bind to lactoferrin in sputum. The binding of omentin to lactoferrin can be increased by galactofuranoside by which omentin interacts with lactoferrin to defend against microbes [89].

### 5.3. Omentin in Obstructive Sleep Apnea Syndrome (OSAS)

Obstructive sleep apnea syndrome (OSAS) is a widespread disease associated with snoring, witnessed apnea, repeated airway obstruction during sleep and excessive daytime sleepiness [101,102], leading to resistance to airflow, oxygen desaturation, hypoxemia and oxidative stress [103]. Studies demonstrated that inflammatory processes, oxidation, and endothelial dysfunction contributed to the occurrence and development of OSAS [104,105]. Importantly, omentin increased endothelial NO expression, mitigated inflammation, and oxidation in human endothelial cells [70,71]. However, studies for determining omentin levels in OSAS patients have yielded contradictory results [61,62,63,106]. Two studies reported that omentin levels increased in patients with OSAS [62,63], whereas the levels of omentin in plasma were reduced to normal values after continuous positive airway pressure (CPAP) therapy for three months, [63]; unfortunately, the sample sizes used in these studies were small. Another study, which had a larger sample size, indicated that omentin levels decreased drastically in OSAS patients and correlated with the severity of OSAS [61]. It also demonstrated that a low omentin level could be regarded as a specific diagnostic marker for the occurrence and deterioration of OSAS [61]. Uygur et al. [106] reported that the lower serum omentin level in OSAS can be reversed by CPAP treatment, which also confirmed the previous observations. More investigations are required to understand the exact roles and mechanisms of omentin in OSAS.

### 5.4. Omentin in Pulmonary Arterial Hypertension (PAH)

Pulmonary arterial hypertension (PAH) is characterized by endothelial dysfunction, inflammation, and vascular remodeling, resulting in increased pulmonary vascular resistance and pulmonary pressure, finally causing right heart failure (RHF) [107]. A series of epidemiological studies highlighted that omentin levels were inversely correlated with obesity, T2DM, and hypertension [16,108]. Moreover, studies showed that omentin levels were extremely low in patients with OSAS and correlated with the severity of OSAS [61,106], which may ultimately induce PAH and RHF. Kazama et al. [72,108] observed that omentin suppressed agonist-induced rise in blood pressure (BP) and monocrotaline-induced enhancement in PA pressure. Additionally, omentin can vasodilate isolated blood vessel via stimulation of endothelium-dependent NO production [109]. 

A previous study revealed that omentin exerted an anti-inflammatory effect on vascular endothelial cells by preventing TNF-α-induced COX-2 expression via inhibiting AMPK/eNOS/NO pathways [70]. Omentin may inhibit expression of the adhesion molecules ICAM-1 and VCAM-1 in endothelial cells [81]. Moreover, omentin played an anti-inflammatory role by inhibiting VCAM-1 in SMCs via inhibition of superoxide production and p38/JNK activation [82]. Importantly, a significant amount of inflammation is necessary for the occurrence and development of structural remodeling in PAH. Kazama et al. [83] also showed that omentin can prevent neointimal hyperplasia via suppression of SMC migration, which was essential for the progression of vascular structural remodeling. Another study also indicated that omentin notably restrained MCT-induced right ventricular hypertrophy [108].

The above evidence suggested that omentin mitigated PAH via suppression of inflammation, SMC migration, and vascular structural remodeling. Thus, omentin possesses potential as a therapeutic tool for PAH and RHF.

### 5.5. Omentin in Acute Respiratory Distress Syndrome (ARDS)

Acute respiratory distress syndrome (ARDS), a devastating disorder distinguished by inflammatory response and endothelial barrier disruption with a 30–60% mortality rate [110,111], is characterized by inflammatory injury, lung edema, and refractory hypoxemia [112]. Studies suggested that omentin can vasodilate isolated blood vessels, suppress the expression of adhesion molecules, and inhibit inflammation in vascular endothelial cells [70,81,109]. Thus, these studies indicated that omentin may play anti-inflammatory and vascular-protective roles in mitigating obesity-related vascular complications.

In addition, direct evidence regarding the impact of omentin on ARDS has also been found. Qi et al. [59] observed that the levels of omentin in ARDS individuals were related to inflammatory responses, and that over-expression of omentin can reduce the expression of IL-6 and TNF-α and attenuate the activation of the NF-κB Rel subunit, thus alleviating pulmonary inflammation in mouse lung tissue. Furthermore, omentin can reduce the levels of IL-6, TNF-α, and VCAM-1 by suppressing NF-κB activation in primary ECs isolated from the lungs or human pulmonary microvascular endothelial cells. Furthermore, omentin can improve pulmonary endothelial cell survival and differentiation, protect pulmonary endothelial barrier function, and decrease the pulmonary microvascular permeability in LPS-induced ARDS models. These results demonstrated that omentin protected the pulmonary endothelial barrier and alleviated pulmonary inflammation by activating the PI3K Akt/eNOS-dependent pathway in LPS-induced ARDS mouse [59]. Thus, omentin may be approved as an effective therapeutic tool for ARDS in the future.

### 5.6. Omentin in Chronic Obstructive Pulmonary Disease (COPD)

Progressive airflow obstruction, destruction of lung parenchyma, and lung inflammation are the characteristic features of COPD, which is a common chronic respiratory disease. Convincing evidence suggested that cigarette smoking (CS) is the major risk factor for COPD [113,114]. Carolan et al. used a microarray screen, TaqMan real time-polymerase chain reaction (RT-PCR), and Western blot analysis to determine whether omentin expression decreased in the airway epithelium of healthy smokers, smokers with lone emphysema, and smokers with established COPD [60]. It is well-known that omentin plays a significant role in the identification of bacterial compositions and pathogens because of its affinity for galactofuranosyl residues [12,13]. Since both active and passive smoke exposure is linked to respiratory infections [115], omentin may be useful to detect bacterial infections in smokers/passive smokers with COPD. Extensive investigations are warranted to dissect the precise molecular mechanisms of omentin in smokers and the onset and progression of COPD. 

## 6. Discussion and Conclusions

Fluctuating omentin levels are widely associated with various diseases, including obesity, diabetes, cardiovascular disease, autoimmune disease, various malignant tumors, reproduction system diseases, nervous system diseases, and pulmonary diseases. Omentin can regulate inflammation status, vasomotor and endothelium function, proliferation, apoptosis, and differentiation of cell types via various molecular mechanisms.

Nonetheless, the mechanism via which omentin regulates various physiological and pathological functions is not completely elucidated. At present, the specific receptor for omentin is still unknown, which makes understanding the physiological function of omentin difficult.

Omentin may play significant roles in pulmonary diseases, such as MPM, asthma, OSAS, PAH, ARDS, COPD, and other lung disorders. Omentin in the pleural effusion may be a specific diagnostic marker for MPM, which is conducive for diagnosing MPM in the early stage, extending the median survival of patients with MPM. Omentin, which belongs to the category of “good” adipokine, exerts anti-inflammatory effects in various physiological and pathological processes. However, current studies have shown that omentin may participate in the pathogenesis of asthma. Omentin is essential for the expression of inflammatory factors in lung epithelial cells, which facilitate allergic airway inflammation, and it may be a component of airway mucus that contributes to pathologic mucus formation in asthma. Omentin levels were drastically reduced in patients with OSAS, which correlated with the severity of OSAS. Moreover, omentin can elicit anti-inflammatory and anti-migratory effects and inhibit vascular structural remodeling for PAH and RHF. Furthermore, omentin can protect pulmonary endothelial barrier function and decrease pulmonary microvascular permeability and inflammation in LPS-induced ARDS models. This indicated that omentin may act as an anti-inflammatory therapeutic drug for ARDS. Studies demonstrated that omentin levels decreased in the airway epithelium of healthy smokers, which is a major risk factor for infections and COPD. This suggests that omentin may contribute to defense against respiratory tract infections and COPD.

Extensive investigations are required to explore the effect of omentin in respiratory diseases. Whether omentin can be used for the diagnosis and assessment of intervention outcomes or development of new therapeutic targets requires further investigation. We expect this review to be helpful for a better understanding of the biological role of omentin in respiratory diseases.

## Figures and Tables

**Table 1:** Fluctuation of omentin levels in various diseases.

Diseases		Fluctuation of Omentin Levels in Various Sample	Omentin-1 Concentration (ng/mL) Mean (Range) or Mean ± SD		*p* Value	Ref.
			CON	Diseases		
Obesity-related diseases	Obesity	Serum ↓	370 ± 20	310 ± 20	0.009	[16]
	Pregnant with preexisting obesity	Cord ↓	58.0 ± 6.0	48.3 ± 9.0	>0.05	[19]
		Maternal ↓	19.5 ± 2.3	7.1 ± 0.9	<0.05	
	T2DM	Serum ↓	18.85 ± 3.23	16.12 ± 4.08	<0.05	[20]
	GDM with obesity	Serum ↓	355.94 ± 42.61	216.41 ± 51.33	0.000	[21]
	T2DM with ischemic heart disease	Serum ↓	12.44 ± 2.12	10.31 ± 2.35	0.038	[23]
	T2DM with Diabetic Retinopathy	Serum ↓	208.31 (164.20–251.20)	139.96 (119.28–157.87)	<0.001	[24]
		Vitreous ↓	96.00 (75.24–112.64)	50.36 (39.91–57.73)	<0.001	
	PCOS	Serum ↓	269.7	197.6	0.0073	[25]
			515.9	210.5	<0.001	[26]
			27.6	23.7	0.05	[28]
	Coronary artery disease	Serum ↓	659.39	373.71	<0.001	[29]
			815.3 ± 185.32	518.61 ± 191.10	<0.001	[30]
			34.58 ± 4.23	10.66 ± 3.41	<0.01	[31]
			254.00 ± 72.98	113.08 ± 61.43	<0.0001	[33]
		EAT ↑ (mRNA)	0.76 (0.71–0.89)	1.25 (1.10–2.85)	0.002	[32]
	coronary heart disease	Serum ↓	1.115 ± 0.361	0.718 ± 0.229	0.000	[34]
	Dilated cardiomyopathy	Serum ↓	233.33 ± 58.04	153.00 ± 48.94	<0.01	[35]
	NAFLD	Serum ↑	376 ± 196	460 ± 181	<0.001	[36]
Chronic immune or inflammatory disease	Psoriasis	Serum ↓	26.8 ± 14.2	18.5 ± 13.1	0.0053	[37]
			488.7 ± 190.3	354.2 ± 152.0	0.001	[38]
			143.60 ± 48.97	95.61 ± 44.38	0.001	[39]
	Rheumatoid arthritis	Serum ↓	23.58 (14.60–28.39)	19.98 (11.98–27.21)	>0.01	[41]
	Behcet disease	Serum ↓	12.4 ± 6.24	8.9 ± 4.65	0.035	[42]
	Crohn’s disease	Serum ↓	409.40 ± 215.65	201.29 ± 76.65	<0.0001	[43]
	Ulcerative colitis	Serum ↓	28.62 (24.71–33.21)	14.74 (11.52–18.16)	<0.001	[44]
	Chronic periodontitis	Gingival crevicular fluid ↓	135	45	<0.008	[45]
	Acute pancreatitis	Serum ↑	22.49 ± 1.4	37.79 ± 1.24	<0.01	[46]
	Chronic pancreatitis	Serum ↑	22.49 ± 1.4	49.37 ± 2.82	<0.01	[46]
	Psoriasis arthritis	Serum ↑	4	20.6 (2.8–82.2)	0.01	[47]
	SLE patients with nephritis	Serum ↑	11.42 (1.44–26.35)	30.77 (16.77–37.63)	0.002	[48]
	HALS	Serum ↓	-	-	0.001	[49]
	Temporo-mandibular disorders	Serum ↓	464.8 ± 191.8	413.5 ±145.9	0.072	[50]
Tumor diseases	Renal cell cancer	Serum ↓	9.86 ± 1.44	3.62 ± 0.76	<0.001	[51]
	MPM	tumor tissues ↑	Serial analysis of gene expression (SAGE) >129 flod increase		-	[52]
	Prostate cancer	Serum ↑	373 (207–792)	546.8 (297.1–945.7)	<0.001	[54]
	colon and colorectal cancer	Serum ↑	0.376 (0.155–0.662)	0.618 (0.151–0.758)	<0.001	[56]
	Gastric cancer	Tumor tissues ↑ (mRNA)	qRT-PCR > 6 flod increase		<0.001	[57]
	Pancreatic adenocarcinoma	Serum ↑	1.61 (0.80–4.98)	9.57 (3.62–21.948)	<0.001	[58]
Respiratory diseases	ARDS	Serum ↓	-	-	<0.05	[59]
	Smokers	airway epithelium ↓ (mRNA/protein)	TaqMan RT-PCR and Immunohistochemistry 3.8–14.7 fold decrease		<0.05	[60]
	OSAS (controversial)	Serum ↓	22.62 (18.71–27.21)	11.29 (8.02–15.13)	<0.001	[61]
		Serum ↑	432.0 (155.2–1101.2)	570.8 (288.4–2152.4)	<0.001	[62]
		Serum ↑	9.24 ± 4.85	17.78 ± 7.20	<0.05	[63]
	Asthma	Airway epithelial ↑ (mRNA)	Arrays and PCR 7.6 and 9.5 flod increase		-	[64]
Others	Liver cirrhosis	Portal venous serum ↑	-	-	0.005	[66]
		hepatic venous seru ↑	-	-	0.027	
		systemic venous serum ↑	-	-	0.032	
	Anorexia nervosa	Serum ↑	185.39 ± 13.98	218.53 ± 18.17	<0.0001	[67]
			34.3 ± 2.6	46.1 ± 3.8	<0.0001	[68]
	End stage renal disease in haemodialysis	Serum ↑	357.5 ±147.4	606.6 ±313.0	<0.001	[69]

ARDS, acute respiratory distress syndrome; GDM, gestational diabetes mellitus; HALS, human immunodeficiency virus/highly active anti-retroviral therapy (HIV/HAART)-associated lipodystrophy syndrome; MPM, malignant pleural mesothelioma; NAFLD, nonalcoholic fatty liver disease; OSAS, obstructive sleep apnoea syndrome; PCOS, polycystic ovary syndrome; SD, Standard Deviation; SLE, systemic lupus erythematosus; T2DM, type-2 diabetes mellitus; Ref., Reference; ↑ = increase, ↓ = decrease, “-” =exact data cannot get from the papers.

## Abbreviations

T2DM	type-2 diabetes mellitus
PCOS	polycystic ovary syndrome
CAD	coronary artery disease
EAT	epicardial adipose tissue
NAFLD	nonalcoholic fatty liver disease
SLE	systemic lupus erythematosus
HIV/HAART	human immunodeficiency virus/highly active anti-retroviral therapy
MPM	malignant pleural mesothelioma
ARDS	acute respiratory distress syndrome
OSAS	obstructive sleep apnoea syndrome
COX-2	cyclooxygenase-2
AMPK	Adenosine 5′-monophosphate-activated protein kinase
eNOS	endothelial nitric oxide synthase
JNK	Jun N-Terminal Kinase
SMC	smooth muscle cell
MCP-1	monocyte chemotactic protein-1
CVSMCs	calcifying vascular smooth muscle cells
PI3K/Akt	phosphatidylinositol 3 kinase/protein kinase B
OPG	osteoprotegerin
RANKL	nuclear factor κB ligand
hOB	human osteoblast
NOX	NADPH oxidase
PDGF-BB	platelet-derived growth factor
OVA	ovalbumin
CPAP	continuous positive airway pressure
COPD	chronic obstructive pulmonary disease

## References

[1] Kralisch S., Klein J., Bluher M., Paschke R., Stumvoll M., Fasshauer M. (2005). Therapeutic perspectives of adipocytokines. Expert Opin. Pharmacother..

[2] Fasshauer M., Bluher M. (2015). Adipokines in health and disease. Trends. Pharmacol. Sci..

[3] Bluher M. (2012). Clinical relevance of adipokines. Diabetes Metab. J..

[4] Koerner A., Kratzsch J., Kiess W. (2005). Adipocytokines: Leptin—The classical, resistin—The controversical, adiponectin—The promising, and more to come. Best Pract. Res. Clin. Endocrinol. Metab..

[5] Hotamisligil G.S., Spiegelman B.M. (1994). Tumor necrosis factor α: A key component of the obesity-diabetes link. Diabetes.

[6] Mohamed-Ali V., Goodrick S., Rawesh A., Katz D.R., Miles J.M., Yudkin J.S., Klein S., Coppack S.W. (1997). Subcutaneous adipose tissue releases interleukin-6, but not tumor necrosis factor-α, in vivo. J. Clin. Endocrinol. Metab..

[7] Bruun J.M., Lihn A.S., Verdich C., Pedersen S.B., Toubro S., Astrup A., Richelsen B. (2003). Regulation of adiponectin by adipose tissue-derived cytokines: In vivo and in vitro investigations in humans. Am. J. Physiol. Endocrinol. Metab..

[8] Bianco A., Nigro E., Monaco M.L., Matera M.G., Scudiero O., Mazzarella G., Daniele A. (2017). The burden of obesity in asthma and COPD: Role of adiponectin. Pulm. Pharmacol. Ther..

[9] Tan Y.L., Zheng X.L., Tang C.K. (2015). The protective functions of omentin in cardiovascular diseases. Pulm. Pharmacol. Ther..

[10] Yang R.Z., Lee M.J., Hu H., Pray J., Wu H.B., Hansen B.C. (2006). Identification of omentin as a novel depot-specific adipokine in human adipose tissue: Possible role in modulating insulin action. Am. J. Physiol. Endocrinol. Metab..

[11] Fain J.N., Sacks H.S., Buehrer B., Bahouth S.W., Garrett E., Wolf R.Y. (2008). Identification of omentin mRNA in human epicardial adipose tissue: Comparison to omentin in subcutaneous, internal mammary artery periadventitial and visceral abdominal depots. Int. J. Obes..

[12] Schaffler A., Neumeier M., Herfarth H., Furst A., Scholmerich J., Buchler C. (2005). Genomic structure of human omentin, a new adipocytokine expressed in omental adipose tissue. Biochim. Biophys. Acta.

[13] Komiya T., Tanigawa Y., Hirohashi S. (1998). Cloning of the novel gene intelectin, which is expressed in intestinal paneth cells in mice. Biochem. Biophys. Res. Commun..

[14] Suzuki Y.A., Shin K., Lonnerdal B. (2001). Molecular cloning and functional expression of a human intestinal lactoferrin receptor. Biochemistry.

[15] Havel P.J. (2004). Update on adipocyte hormones: Regulation of energy balance and carbohydrate/lipid metabolism. Diabetes.

[16] De Souza Batista C.M., Yang R.Z., Lee M.J., Glynn N.M., Yu D.Z., Pray J., Ndubuizu K., Patil S., Schwartz A., Kligman M. (2007). Omentin plasma levels and gene expression are decreased in obesity. Diabetes.

[17] Bonet M.L., Oliver P., Palou A. (2013). Pharmacological and nutritional agents promoting browning of white adipose tissue. Biochim. Biophys. Acta.

[18] Olarescu N.C., Heck A., Godang K., Ueland T., Bollerslev J. (2016). The Metabolic risk in patients newly diagnosed with acromegaly is related to fat distribution and circulating adipokines and improves after treatment. Neuroendocrinology.

[19] Barker G., Lim R., Georgiou H.M., Lappas M. (2012). Omentin-1 is decreased in maternal plasma, placenta and adipose tissue of women with pre-existing obesity. PLoS ONE.

[20] Pan H.Y., Guo L., Li Q. (2010). Changes of serum omentin-1 levels in normal subjects and in patients with impaired glucose regulation and with newly diagnosed and untreated type 2 diabetes. Diabetes. Res. Clin. Pract..

[21] Pan B.L., Ma R.M. (2016). Correlation of serum omentin-1 and chemerin with gestational diabetes mellitus. J. South. Med. Univ..

[22] Pourbehi M.R., Zahedi T., Darabi H., Ostovar A., Assadi M., Nabipour I. (2016). Omentin-1 and nonfatal ischemic heart disease in postmenopausal women: A population-based study. Endocr. Pract..

[23] Alkuraishy H.M., Al-Gareeb A.I. (2015). New insights into the role of metformin effects on serum omentin-1 levels in acute myocardial infarction: Cross-sectional study. Emerg. Med. Int..

[24] Wan W., Li Q., Zhang F., Zheng G., Lv Y., Wan G., Jin X. (2015). Serum and vitreous concentrations of omentin-1 in diabetic retinopathy. Dis. Markers.

[25] Kort D.H., Kostolias A., Sullivan C., Lobo R.A. (2015). Chemerin as a marker of body fat and insulin resistance in women with polycystic ovary syndrome. Gynecol. Endocrinol..

[26] Orlik B., Madej P., Owczarek A., Skalba P., Chudek J., Olszanecka-Glinianowicz M. (2014). Plasma omentin and adiponectin levels as markers of adipose tissue dysfunction in normal weight and obese women with polycystic ovary syndrome. Clin. Endocrinol..

[27] Tang Y.L., Yu J., Zeng Z.G., Liu Y., Liu J.Y., Xu J.X. (2017). Circulating omentin-1 levels in women with polycystic ovary syndrome: A meta-analysis. Gynecol. Endocrinol..

[28] Tan B.K., Adya R., Farhatullah S., Chen J., Lehnert H., Randeva H.S. (2010). Metformin treatment may increase omentin-1 levels in women with polycystic ovary syndrome. Diabetes.

[29] Du Y., Ji Q., Cai L., Huang F., Lai Y., Liu Y., Yu J., Han B., Zhu E., Zhang J. (2016). Association between omentin-1 expression in human epicardial adipose tissue and coronary atherosclerosis. Cardiovasc. Diabetol..

[30] Kadoglou N.P., Lambadiari V., Gastounioti A., Gkekas C., Giannakopoulos T.G., Koulia K., Maratou E., Alepaki M., Kakisis J., Karakitsos P. (2014). The relationship of novel adipokines, RBP4 and omentin-1, with carotid atherosclerosis severity and vulnerability. Atherosclerosis.

[31] Liu R., Wang X., Bu P. (2011). Omentin-1 is associated with carotid atherosclerosis in patients with metabolic syndrome. Diabetes. Res. Clin. Pract..

[32] Harada K., Shibata R., Ouchi N., Tokuda Y., Funakubo H., Suzuki M., Kataoka T., Nagao T., Okumura S., Shinoda N. (2016). Increased expression of the adipocytokine omentin in the epicardial adipose tissue of coronary artery disease patients. Atherosclerosis.

[33] Zhong X., Zhang H.Y., Tan H., Zhou Y., Liu F.L., Chen F.Q., Yu J., Han B., Zhu E., Zhang J. (2011). Association of serum omentin-1 levels with coronary artery disease. Acta Pharmacol. Sin..

[34] Wang X.H., Dou L.Z., Gu C., Wang X.Q. (2014). Plasma levels of omentin-1 and visfatin in senile patients with coronary heart disease and heart failure. Asian Pac. J. Trop. Med..

[35] Huang Y., Lin Y., Zhang S., Wang Z., Zhang J., Chang C., Liu L., Ji Q., Liu X. (2016). Circulating omentin-1 levels are decreased in dilated cardiomyopathy patients with overt heart failure. Dis. Markers.

[36] Yilmaz Y., Yonal O., Kurt R., Alahdab Y.O., Eren F., Ozdogan O., Celikel C.A., Imeryuz N., Kalayci C., Avsar E. (2011). Serum levels of omentin, chemerin and adipsin in patients with biopsy-proven nonalcoholic fatty liver disease. Scand. J. Gastroenterol..

[37] Ismail S.A., Mohamed S.A. (2012). Serum levels of visfatin and omentin-1 in patients with psoriasis and their relation to disease severity. Br. J. Dermatol..

[38] Turan H., Yaykasli K.O., Soguktas H., Yaykasli E., Aliagaoglu C., Erdem T., Karkucak M., Kaya E., Ucgun T., Bahadir A. (2014). Omentin serum levels and omentin gene Val109Asp polymorphism in patients with psoriasis. Int. J. Dermatol..

[39] Zhang C., Zhu K.J., Liu J.L., Xu G.X., Liu W., Jiang F.X., Zheng H.F., Quan C. (2015). Omentin-1 plasma levels and omentin-1 expression are decreased in psoriatic lesions of psoriasis patients. Arch. Dermatol. Res..

[40] Robinson C., Tsang L., Solomon A., Woodiwiss A.J., Gunter S., Millen A.M., Norton G.R., Fernandez-Lopez M.J., Hollan I., Dessein P.H. (2017). Omentin concentrations are independently associated with those of matrix metalloproteinase-3 in patients with mild but not severe rheumatoid arthritis. Rheumatol. Int..

[41] Xu L., Zhu G.B., Wang L., Wang D.F., Jiang X.R. (2012). Synovial fluid omentin-1 levels are inversely correlated with radiographic severity of knee osteoarthritis. J. Investig. Med..

[42] Turkcu F.M., Sahin A., Cingu A.K., Kaya S., Yuksel H., Cinar Y., Batmaz İ. (2015). Serum omentin, resistin and tumour necrosis factor-α levels in Behcet patients with and without ocular involvement. Graefes. Arch. Clin. Exp. Ophthalmol..

[43] Lu Y., Zhou L., Liu L., Feng Y., Lu L., Ren X., Dong X., Sang W. (2014). Serum omentin-1 as a disease activity marker for Crohn’s disease. Dis. Markers.

[44] Yin J., Hou P., Wu Z., Nie Y. (2015). Decreased levels of serum omentin-1 in patients with inflammatory bowel disease. Med. Sci. Monit..

[45] Bozkurt Dogan S., Ongoz Dede F., Balli U., Sertoglu E. (2016). Levels of vaspin and omentin-1 in gingival crevicular fluid as potential markers of inflammation in patients with chronic periodontitis and type 2 diabetes mellitus. J. Oral. Sci..

[46] Sit M., Aktas G., Yilmaz E.E., Alcelik A., Terzi E.H., Tosun M. (2014). Effects of the inflammatory response on serum omentin levels in early acute and chronic pancreatitis. Clin. Ter..

[47] Xue Y., Jiang L., Cheng Q., Chen H., Yu Y., Lin Y., Yang X., Kong N., Zhu X., Xu X. (2012). Adipokines in psoriatic arthritis patients: The correlations with osteoclast precursors and bone erosions. PLoS ONE.

[48] Zhang T.P., Li H.M., Leng R.X., Li X.P., Li X.M., Pan H.F., Ye D.Q. (2016). Plasma levels of adipokines in systemic lupus erythematosus patients. Cytokine.

[49] Peraire J., Lopez-Dupla M., Alba V., Beltran-Debon R., Martinez E., Domingo P., Asensi V., Leal M., Viladés C., Inza M.I. (2015). HIV/antiretroviral therapy-related lipodystrophy syndrome (HALS) is associated with higher RBP4 and lower omentin in plasma. Clin. Microbiol. Infect..

[50] Harmon J.B., Sanders A.E., Wilder R.S., Essick G.K., Slade G.D., Hartung J.E., Nackley A.G. (2016). Circulating omentin-1 and chronic painful temporomandibular disorders. J. Oral Facial Pain Headache.

[51] Shen X.D., Zhang L., Che H., Zhang Y.Y., Yang C., Zhou J., Liang C.Z. (2016). Circulating levels of adipocytokine omentin-1 in patients with renal cell cancer. Cytokine.

[52] Wali A., Morin P.J., Hough C.D., Lonardo F., Seya T., Carbone M., Pass H.I. (2005). Identification of intelectin overexpression in malignant pleural mesothelioma by serial analysis of gene expression (SAGE). Lung Cancer.

[53] Zhang Y.Y., Zhou L.M. (2013). Omentin-1, a new adipokine, promotes apoptosis through regulating Sirt1-dependent p53 deacetylation in hepatocellular carcinoma cells. Eur. J. Pharmacol..

[54] Uyeturk U., Sarici H., Kin Tekce B., Eroglu M., Kemahli E., Gucuk A. (2014). Serum omentin level in patients with prostate cancer. Med. Oncol..

[55] Fazeli M.S., Dashti H., Akbarzadeh S., Assadi M., Aminian A., Keramati M.R., Nabipour I. (2013). Circulating levels of novel adipocytokines in patients with colorectal cancer. Cytokine.

[56] Uyeturk U., Alcelik A., Aktas G., Tekce B.K. (2014). Post-treatment plasma omentin levels in patients with stage III colon carcinoma. J. BUON.

[57] Zheng L., Weng M., Qi M., Qi T., Tong L., Hou X., Tong Q. (2012). Aberrant expression of intelectin-1 in gastric cancer: Its relationship with clinicopathological features and prognosis. J. Cancer Res. Clin. Oncol..

[58] Karabulut S., Afsar C.U., Karabulut M., Alis H., Bozkurt M.A., Aydogan F., Serilmez M., Tas F. (2016). Clinical significance of serum omentin-1 levels in patients with pancreatic adenocarcinoma. BBA Clin..

[59] Qi D., Tang X., He J., Wang D., Zhao Y., Deng W., Deng X., Zhou G., Xia J., Zhong X. (2016). Omentin protects against LPS-induced ARDS through suppressing pulmonary inflammation and promoting endothelial barrier via an Akt/eNOS-dependent mechanism. Cell Death Dis..

[60] Carolan B.J., Harvey B.G., De B.P., Vanni H., Crystal R.G. (2008). Decreased expression of intelectin 1 in the human airway epithelium of smokers compared to nonsmokers. J. Immunol..

[61] Wang Q., Feng X., Zhou C., Li P., Kang J. (2013). Decreased levels of serum omentin-1 in patients with obstructive sleep apnoea syndrome. Ann. Clin. Biochem..

[62] Kurt O.K., Tosun M., Alcelik A., Yilmaz B., Talay F. (2014). Serum omentin levels in patients with obstructive sleep apnea. Sleep Breath..

[63] Zirlik S., Hildner K.M., Targosz A., Neurath M.F., Fuchs F.S., Brzozowski T., Konturek P.C. (2013). Melatonin and omentin: Influence factors in the obstructive sleep apnoea syndrome?. J. Physiol. Pharmacol..

[64] Kuperman D.A., Lewis C.C., Woodruff P.G., Rodriguez M.W., Yang Y.H., Dolganov G.M., Fahy J.V., Erle D.J. (2005). Dissecting asthma using focused transgenic modeling and functional genomics. J. Allergy Clin. Immunol..

[65] Wang C. (2014). Obesity, inflammation, and lung injury (OILI): The good. Mediat. Inflamm..

[66] Eisinger K., Krautbauer S., Wiest R., Karrasch T., Hader Y., Scherer M.N., Farkas S., Aslanidis C., Buechler C. (2013). Portal vein omentin is increased in patients with liver cirrhosis but is not associated with complications of portal hypertension. Eur. J. Clin. Investig..

[67] Guo L.J., Jiang T.J., Liao L., Liu H., He H.B. (2013). Relationship between serum omentin-1 level and bone mineral density in girls with anorexia nervosa. J. Endocrinol. Investig..

[68] Oswiecimska J., Suwala A., Swietochowska E., Ostrowska Z., Gorczyca P., Ziora-Jakutowicz K., Machura E., Szczepańska M., Kukla M., Stojewska M. (2015). Serum omentin levels in adolescent girls with anorexia nervosa and obesity. Physiol. Res..

[69] Alcelik A., Tosun M., Ozlu M.F., Eroglu M., Aktas G., Kemahli E., Savli H., Yazici M. (2012). Serum levels of omentin in end-stage renal disease patients. Kidney Blood Press. Res..

[70] Yamawaki H., Kuramoto J., Kameshima S., Usui T., Okada M., Hara Y. (2011). Omentin, a novel adipocytokine inhibits TNF-induced vascular inflammation in human endothelial cells. Biochem. Biophys. Res. Commun..

[71] Maruyama S., Shibata R., Kikuchi R., Izumiya Y., Rokutanda T., Araki S., Kataoka Y., Ohashi K., Daida H., Kihara S. (2012). Fat-derived factor omentin stimulates endothelial cell function and ischemia-induced revascularization via endothelial nitric oxide synthase-dependent mechanism. J. Biol. Chem..

[72] Kazama K., Okada M., Hara Y., Yamawaki H. (2013). A novel adipocytokine, omentin, inhibits agonists-induced increases of blood pressure in rats. J. Vet. Med. Sci..

[73] Brunetti L., Leone S., Orlando G., Ferrante C., Recinella L., Chiavaroli A., Di Nisio C., Shohreh R., Manippa F., Ricciuti A. (2014). Hypotensive effects of omentin-1 related to increased adiponectin and decreased interleukin-6 in intra-thoracic pericardial adipose tissue. Pharmacol. Rep..

[74] Matsuo K., Shibata R., Ohashi K., Kambara T., Uemura Y., Hiramatsu-Ito M., Enomoto T., Yuasa D., Joki Y., Ito M. (2015). Omentin functions to attenuate cardiac hypertrophic response. J. Mol. Cell. Cardiol..

[75] Watanabe K., Watanabe R., Konii H., Shirai R., Sato K., Matsuyama T.A., Ishibashi-Ueda H., Koba S., Kobayashi Y., Hirano T. (2016). Counteractive effects of omentin-1 against atherogenesisdagger. Cardiovasc. Res..

[76] Kataoka Y., Shibata R., Ohashi K., Kambara T., Enomoto T., Uemura Y., Ogura Y., Yuasa D., Matsuo K., Nagata T. (2014). Omentin prevents myocardial ischemic injury through AMP-activated protein kinase and Akt-dependent mechanisms. J. Am. Coll. Cardiol..

[77] Hiramatsu-Ito M., Shibata R., Ohashi K., Uemura Y., Kanemura N., Kambara T., Enomoto T., Yuasa D., Matsuo K., Ito M. (2016). Omentin attenuates atherosclerotic lesion formation in apolipoprotein E-deficient mice. Cardiovasc. Res..

[78] Duan X.Y., Xie P.L., Ma Y.L., Tang S.Y. (2011). Omentin inhibits osteoblastic differentiation of calcifying vascular smooth muscle cells through the PI3K/Akt pathway. Amino Acids.

[79] Xie H., Xie P.L., Wu X.P., Chen S.M., Zhou H.D., Yuan L.Q., Sheng Z.F., Tang S.Y., Luo X.H., Liao E.Y. (2011). Omentin-1 attenuates arterial calcification and bone loss in osteoprotegerin-deficient mice by inhibition of RANKL expression. Cardiovasc. Res..

[80] Wu S.S., Liang Q.H., Liu Y., Cui R.R., Yuan L.Q., Liao E.Y. (2013). Omentin-1 stimulates human osteoblast proliferation through PI3K/Akt signal pathway. Int. J. Endocrinol..

[81] Zhong X., Li X., Liu F., Tan H., Shang D. (2012). Omentin inhibits TNF-α-induced expression of adhesion molecules in endothelial cells via ERK/NF-κB pathway. Biochem. Biophys. Res. Commun..

[82] Kazama K., Usui T., Okada M., Hara Y., Yamawaki H. (2012). Omentin plays an anti-inflammatory role through inhibition of TNF-α-induced superoxide production in vascular smooth muscle cells. Eur. J. Pharmacol..

[83] Kazama K., Okada M., Yamawaki H. (2014). A novel adipocytokine, omentin, inhibits platelet-derived growth factor-BB-induced vascular smooth muscle cell migration through antioxidative mechanism. Am. J. Physiol. Heart Circ. Physiol..

[84] Chailleux E., Dabouis G., Pioche D., de Lajartre M., de Lajartre A.Y., Rembeaux A., Germaud P. (1988). Prognostic factors in diffuse malignant pleural mesothelioma. A study of 167 patients. Chest.

[85] Ruffie P., Feld R., Minkin S., Cormier Y., Boutan-Laroze A., Ginsberg R., Ayoub J., Shepherd F.A., Evans W.K., Figueredo A. (1989). Diffuse malignant mesothelioma of the pleura in Ontario and Quebec: A retrospective study of 332 patients. J. Clin. Oncol..

[86] Tsuji S., Tsuura Y., Morohoshi T., Shinohara T., Oshita F., Yamada K., Kameda Y., Ohtsu T., Nakamura Y., Miyagi Y. (2010). Secretion of intelectin-1 from malignant pleural mesothelioma into pleural effusion. Br. J. Cancer.

[87] Washimi K., Yokose T., Yamashita M., Kageyama T., Suzuki K., Yoshihara M., Miyagi Y., Hayashi H., Tsuji S. (2012). Specific expression of human intelectin-1 in malignant pleural mesothelioma and gastrointestinal goblet cells. PLoS ONE.

[88] Zhen G., Park S.W., Nguyenvu L.T., Rodriguez M.W., Barbeau R., Paquet A.C., Erle D.J. (2007). IL-13 and epidermal growth factor receptor have critical but distinct roles in epithelial cell mucin production. Am. J. Respir. Cell Mol. Biol..

[89] Kerr S.C., Carrington S.D., Oscarson S., Gallagher M.E., Solon M., Yuan S., Ahn J.N., Dougherty R.H., Finkbeiner W.E., Peters M.C. (2014). Intelectin-1 is a prominent protein constituent of pathologic mucus associated with eosinophilic airway inflammation in asthma. Am. J. Respir. Crit. Care Med..

[90] Pemberton A.D., Rose-Zerilli M.J., Holloway J.W., Gray R.D., Holgate S.T. (2008). A single-nucleotide polymorphism in intelectin 1 is associated with increased asthma risk. J. Allergy Clin. Immunol..

[91] Wills-Karp M., Luyimbazi J., Xu X., Schofield B., Neben T.Y., Karp C.L., Donaldson D.D. (1998). Interleukin-13: Central mediator of allergic asthma. Science.

[92] Grunig G., Warnock M., Wakil A.E., Venkayya R., Brombacher F., Rennick D.M., Sheppard D., Mohrs M., Donaldson D.D., Locksley R.M. (1998). Requirement for IL-13 independently of IL-4 in experimental asthma. Science.

[93] Ip W.K., Wong C.K., Lam C.W. (2006). Interleukin (IL)-4 and IL-13 up-regulate monocyte chemoattractant protein-1 expression in human bronchial epithelial cells: Involvement of p38 mitogen-activated protein kinase, extracellular signal-regulated kinase 1/2 and Janus kinase-2 but not c-Jun NH2-terminal kinase 1/2 signalling pathways. Clin. Exp. Immunol..

[94] Gu N., Kang G., Jin C., Xu Y., Zhang Z., Erle D.J., Zhen G. (2010). Intelectin is required for IL-13-induced monocyte chemotactic protein-1 and -3 expression in lung epithelial cells and promotes allergic airway inflammation. Am. J. Physiol. Lung Cell. Mol. Physiol..

[95] Tsuji S., Uehori J., Matsumoto M., Suzuki Y., Matsuhisa A., Toyoshima K., Seya T. (2001). Human intelectin is a novel soluble lectin that recognizes galactofuranose in carbohydrate chains of bacterial cell wall. J. Biol. Chem..

[96] Lambrecht B.N., Hammad H. (2013). Asthma: The importance of dysregulated barrier immunity. Eur. J. Immunol..

[97] Licona-Limon P., Kim L.K., Palm N.W., Flavell R.A. (2013). TH2, allergy and group 2 innate lymphoid cells. Nat. Immunol..

[98] Yi L., Cheng D., Zhang K., Huo X., Mo Y., Shi H., Di H., Zou Y., Zhang H., Zhao J. (2017). Intelectin contributes to allergen-induced IL-25, IL-33, and TSLP expression and type 2 response in asthma and atopic dermatitis. Mucosal Immunol..

[99] Pemberton A.D., Verdon B., Inglis N.F., Pearson J.P. (2011). Sheep intelectin-2 co-purifies with the mucin Muc5ac from gastric mucus. Res. Vet. Sci..

[100] Morcillo E.J., Cortijo J. (2006). Mucus and MUC in asthma. Curr. Opin. Pulm. Med..

[101] Azagra-Calero E., Espinar-Escalona E., Barrera-Mora J.M., Llamas-Carreras J.M., Solano-Reina E. (2012). Obstructive sleep apnea syndrome (OSAS). Review of the literature. Med Oral Patol. Oral Cir. Bucal.

[102] Hecht L., Mohler R., Meyer G. (2011). Effects of CPAP-respiration on markers of glucose metabolism in patients with obstructive sleep apnoea syndrome: A systematic review and meta-analysis. Ger. Med. Sci..

[103] Franco C.M., Lima A.M., Ataide L., Lins O.G., Castro C.M., Bezerra A.A., de Oliveira M.F., Oliveira J.R. (2012). Obstructive sleep apnea severity correlates with cellular and plasma oxidative stress parameters and affective symptoms. J. Mol. Neurosci..

[104] Jelic S., Padeletti M., Kawut S.M., Higgins C., Canfield S.M., Onat D., Colombo P.C., Basner R.C., Factor P., LeJemtel T.H. (2008). Inflammation, oxidative stress, and repair capacity of the vascular endothelium in obstructive sleep apnea. Circulation.

[105] El Solh A.A., Akinnusi M.E., Baddoura F.H., Mankowski C.R. (2007). Endothelial cell apoptosis in obstructive sleep apnea: A link to endothelial dysfunction. Am. J. Respir. Crit. Care Med..

[106] Uygur F., Tanriverdi H., Can M., Erboy F., Altinsoy B., Atalay F., Ornek T., Damar M., Kokturk F., Tor M. (2016). Association between continuous positive airway pressure and circulating omentin levels in patients with obstructive sleep apnoea. Sleep Breath..

[107] Lai Y.C., Potoka K.C., Champion H.C., Mora A.L., Gladwin M.T. (2014). Pulmonary arterial hypertension: The clinical syndrome. Circ. Res..

[108] Kazama K., Okada M., Yamawaki H. (2014). A novel adipocytokine, omentin, inhibits monocrotaline-induced pulmonary arterial hypertension in rats. Biochem. Biophys. Res. Commun..

[109] Yamawaki H., Tsubaki N., Mukohda M., Okada M., Hara Y. (2010). Omentin, a novel adipokine, induces vasodilation in rat isolated blood vessels. Biochem. Biophys. Res. Commun..

[110] Ranieri V.M., Rubenfeld G.D. (2012). Thompson, B.T.; Ferguson, N.D.; Caldwell, E.; Fan, E.; Camporota, L.; Slutsky, A.S. Acute respiratory distress syndrome: The Berlin Definition. JAMA.

[111] Gando S., Kameue T., Matsuda N., Sawamura A., Hayakawa M., Kato H. (2004). Systemic inflammation and disseminated intravascular coagulation in early stage of ALI and ARDS: Role of neutrophil and endothelial activation. Inflammation.

[112] Duan J.X., Zhou Y., Zhou A.Y., Guan X.X., Liu T., Yang H.H., Xie H., Chen P. (2017). Calcitonin gene-related peptide exerts anti-inflammatory property through regulating murine macrophages polarization in vitro. Mol. Immunol..

[113] Vestbo J., Hurd S.S., Agusti A.G., Jones P.W., Vogelmeier C., Anzueto A. (2013). Global strategy for the diagnosis, management, and prevention of chronic obstructive pulmonary disease: GOLD executive summary. Am. J. Respir. Crit. Care Med..

[114] Zuo L., He F., Sergakis G.G., Koozehchian M.S., Stimpfl J.N., Rong Y., Diaz P.T., Best T.M. (2014). Interrelated role of cigarette smoking, oxidative stress, and immune response in COPD and corresponding treatments. Am. J. Physiol. Lung Cell. Mol. Physiol..

[115] Murin S., Bilello K.S. (2005). Respiratory tract infections: Another reason not to smoke. Clevel. Clin. J. Med..

